# Very Early PSA Response to Abiraterone in mCRPC Patients: A Novel Prognostic Factor Predicting Overall Survival

**DOI:** 10.3389/fphar.2016.00123

**Published:** 2016-05-18

**Authors:** Gaetano Facchini, Orazio Caffo, Cinzia Ortega, Carmine D'Aniello, Marilena Di Napoli, Sabrina C. Cecere, Chiara Della Pepa, Anna Crispo, Francesca Maines, Fiorella Ruatta, Gelsomina Iovane, Salvatore Pisconti, Maurizio Montella, Massimiliano Berretta, Sandro Pignata, Carla Cavaliere

**Affiliations:** ^1^Division of Medical Oncology, Department of Uro-Gynaecological Oncology, Istituto Nazionale Tumori “Fondazione G. Pascale” - IRCCSNaples, Italy; ^2^Department of Medical Oncology, Santa Chiara HospitalTrento, Italy; ^3^Medical Oncology Department, Institute for Cancer Research and TreatmentCandiolo, Italy; ^4^Department of Onco-Ematology Medical Oncology, S.G. Moscati Hospital of TarantoTaranto, Italy; ^5^Unit of Epidemiology, Struttura Complessa di Statistica Medica, Biometria e Bioinformatica, Fondazione IRCCS Istituto Nazionale TumoriNaples, Italy; ^6^Department of Medical Oncology, National Cancer InstituteAviano, Italy

**Keywords:** prostate cancer, prognostic factors, PSA detection, metastatic castration resistant prostate cancer, abiraterone acetato, overall survival

## Abstract

**Background:** Abiraterone Acetate (AA) is approved for the treatment of mCRPC after failure of androgen deprivation therapy in whom chemotherapy is not yet clinically indicated and for treatment of mCRPC progressed during or after docetaxel-based chemotherapy regimen. The aim of this study is to evaluate the role of early PSA decline for detection of therapy success or failure in mCRPC patients treated with AA in post chemotherapy setting.

**Patients and Methods:** We retrospectively evaluated 87 patients with mCRPC treated with AA. Serum PSA levels were evaluated after 15, 90 days and then monthly. The PSA flare phenomenon was evaluated, according to a confirmation value at least 1 week apart. The primary endpoint was to demonstrate that an early PSA decline correlates with a longer progression free survival (PFS) and overall survival (OS). The secondary endpoind was to demonstrate a correlation between better outcome and demographic and clinical patient characteristics.

**Results:** We have collected data of 87 patients between Sep 2011 and Sep 2014. Early PSA response (≥50% from baseline at 15 days) was found in 56% evaluated patients and confirmed in 29 patients after 90 days. The median PFS was 5.5 months (4.6–6.5) and the median OS was 17.1 months (8.8–25.2). In early responders patients (PSA RR ≥ 50% at 15 days), we found a significant statistical advantage in terms of PFS at 1 year, HR 0.28, 95%CI 0.12–0.65, *p* = 0.003, and OS, HR 0.21 95% CI 0.06–0.72, *p* = 0.01. The results in PFS at 1 years and OS reached statistical significance also in the evaluation at 90 days.

**Conclusion:** A significant proportion (78.6%) of patients achieved a rapid response in terms of PSA decline. Early PSA RR (≥50% at 15 days after start of AA) can provide clinically meaningful information and can be considered a surrogate of longer PFS and OS.

## Introduction

Prostate cancer growth depend on signaling from the activated androgen receptor (AR). Nearly 90% of all patients with metastatic prostate cancer initially respond to castration-induced androgen deprivation. Unfortunately, this therapeutic response is not lasting, with a median duration of 18–24 months, after which the tumor progresses despite hormonal castration levels, so we define a prostate cancer as castration-resistant (CRPC). Some authors reported that the AR remains activated and plays an important role during the progression of metastatic CRPC (mCRPC; Zhu et al., 2010; D'Aniello et al., 2014; Zhang et al., 2015). Several mechanisms are involved such as: genomic amplification and over expression of AR, gain of function mutations of AR, up-regulation of AR enhancer elements, alterations in androgen transport, increased synthesis of extra gonadal androgens, abnormalities in AR co-activators and co-regulators, ligand-independent trans-activation of AR by growth factors or cytokines, and AR splice variants (Rathkopf and Scher, 2013). Docetaxel was the first systemic therapy to show an improvement in overall survival (OS) in patients with mCRPC (Petrylak et al., 2004; Tannock et al., 2004). Recently, different agents with different mechanisms of action have demonstrated efficacy, and four new drugs (cabazitaxel [CBZ], sipuleucel-T, enzalutamide [ENZ], and abiraterone acetate [AA]) were approved for mCRPC treatment (de Bono et al., 2010, 2011; Kantoff et al., 2010; Fizazi et al., 2012; Scher et al., 2012; Bahl et al., 2013; Di Lorenzo et al., 2013). Among these, AA is an inhibitor of the androgen biosynthesis enzyme CYP17 (17-a-hydroxylase and C17, 20-lyase) and it has been approved for treatment of mCRPC after failure of androgen deprivation therapy in whom chemotherapy is not yet clinically indicated and for the treatment of mCRPC progressed on or after a docetaxel-based chemotherapy regimen (de Bono et al., 2011; Fizazi et al., 2012).

The COU-AA 301 trials demonstrated a prolonged OS in AA–prednisone group than in the placebo–prednisone group. Median time to PSA progression was 8.5 months in the AA group vs. 6.6 months in the placebo group with a confirmed advantage in the AA treatment (Marra et al., 2008; Facchini et al., 2010; de Bono et al., 2011; Fizazi et al., 2012; Zhou et al., 2014).

In this context, it is required the validation of predictive markers of response. PSA decline after cytotoxic chemotherapy has been identified as a valid surrogate for OS and PSA progression-free survival at 3 months, in fact its reductions may reflect reductions in tumor burden. Many retrospective reports confirmed that patients with mCRPC who had a 50% decline in PSA from baseline, had a survival improvement, compared with patients who did not achieve 50% reduction in PSA (Smith et al., 1998; Scher et al., 1999; Small et al., 2002; Halabi et al., 2013). However, to date, its role as surrogate OS endpoints remains uncertain, especially in the course of non-cytotoxic therapies such as new-generation hormonal agents for still few data in this setting. Recent evidences demonstrate that tubulin-targeting drugs (docetaxel) cause cytoplasmic AR sequestration *ex vivo* (Jiang and Huang, 2010; Zhu et al., 2010; Buonerba et al., 2011; Mezynski et al., 2012) and in circulating tumor cells (Buonerba et al., 2011), significant down-regulation of AR and PSA expression, and nuclear accumulation of the fork head transcription factor family member FOXO1. This last is a potent repressor of AR function (Gan et al., 2009; Kuroda et al., 2009; Franco et al., 2011) and suggest the strong evidence that the antitumor activity of docetaxel is also associated with disruption of AR signaling. These data could support the hypothesis of a cross-resistance between docetaxel and AA. The activity of AA, similarly, also appears to differ according to its sequencing with docetaxel (Caraglia et al., 2005; Mezynski et al., 2012; Loriot et al., 2013). The aim of our retrospective analysis is to demonstrate that an early PSA decline correlates with longer progression free survival (PFS) and OS in order to validate this parameter as an early predictive marker of AA response, in order to exclude from treatment patients non-responders, therefore reducing costs, and serial determinations of PSA.

## Patients and methods

This retrospective study enrolled 87 patients at three sites in Italy (National Cancer Institute of Naples–Fondazione G. Pascale, Oncological Institute of Candiolo and Santa Chiara Hospital in Trento) with histologically confirmed metastatic prostate cancer, who became refractory to hormonal therapy, and progressed after chemotherapy (treated with at least one line docetaxel containing regimen). All patients received AA (1000 mg orally once daily at least 1 h before or 2 h after meal, with prednisone 10 mg daily). Each cycle of treatment was 28 days long. All patients maintained androgen deprivation with a serum testosterone level of 50 ng per decilitre or less (≤2.0 nmol per liter). During AA treatment, patients monthly had a check of their hematological parameters. PSA value was recorded after 15 days from the start of therapy and then monthly. Tumor assessment with computed tomography (CT) and/or bone scan was performed at baseline and then every 3 months or as clinical indicated. Baseline ECG and Echocardiogram were obtained and further cardiac work-up was performed if indicated. Treatment could be continued until disease progression, which was defined based on the Prostate Cancer Working Group II criteria, death or unacceptable toxicity. In case of biochemical progression only during the first 3 months of treatment, evidence of radiological/metabolic progression was required to stop the therapy. Patients were stratified according to variables considered potential predictors of better outcome included: baseline ECOG performance status score (0 or 1 vs. 2), age (≤70 vs. >70 years), Gleason score (≤7 vs. >7), previous prostatectomy (yes or no), previous curative radiotherapy (yes or no), duration of previous ADT for metastatic disease, number of previous chemotherapy regimens (1 vs. >1), extent of disease (visceral vs. non-visceral disease), previous cumulative docetaxel dose expressed in mg/m^2^ (≤675 vs. >675 mg/m^2^), duration of AA treatment. Table 1 summarizes Baseline Demographic and Clinical Patient Characteristics.

**Table 1:** **Baseline Demographic and Clinical Patients' Characteristics**.

**All subjects**	**87 N. pts/total (%)**
**AGE**	
Median (range) − years 72.2 (48–84)	
>70 years	52/87 (59.8)
**ECOG**	
0–1	75/87 (86.2)
>1	12/87 (13.8)
**GLEASON**	
≤7	43/87 (34.5)
>7	48/87 (55.1)
Missing data	9/87 (10.3)
**PREVIOUS THERAPIES**	
**Prostatectomy**	
Yes	41/87 (47.1)
No	46/87 (52.9)
**CURATIVE RADIOTHERAPY**	
Yes	24/87 (27.6)
No	59/87 (67.8)
Missing data	4/87 (4.6)
**DURATION OF PREVIOUS ADT FOR METASTATIC DISEASE**	
<2.5 years	43/87 (49.4)
≥2.5 years	41/87 (47.1)
Missing data	3/87 (3.4)
**PREVIOUS CYTOTOXIC CHEMOTHERAPY**	
1	60/87 (68.9)
>1	27/87 (31.1)
**TOTAL PREVIOUS DOCETAXEL DOSE (mg/m**^2^**)**	
≤675 mg/m^2^	50/87 (57.5)
>675 mg/m^2^	36/87 (41.4)
Missing data	1/87 (1.1)
**TREATMENT DURATION WITH AA**	
≤7 months	54/87 (62.1)
>7 months	22/87 (25.3)
Missing data	11/87 (12.6)
**EXTENT OF DISEASE**	
No visceral disease	79/87 (90.8)
Visceral disease	8/87 (9.1)

Early PSA response rate (PSA RR) was assessed 15 days after the start of treatment, and then after 12 weeks (according with PCWG 2). Early PSA RR (at 15 days) was defined as a ≥50% reduction from baseline, stratifying patients as early responders or early non-responders PSA and PSA Progression for an increase of 25% or 5 ng/ml absolute value from baseline (Table 2). Our aim is to demonstrate that an early PSA decline correlate with longer PFS and OS in order to validate this parameter as an early predictive marker of response to AA, excluding patients non-responders and therefore reducing costs treatment related and serial determinations of PSA.

**Table 2:** **PSA value and response**.

**PSA**	
Median baseline (range)—ng/dl	332.2 (1–3922)
**PSA RR at 15 days-**	**N. pts/total (%)**
No of patient evaluable	75/87
≥50%	42/75 (56)
<50%	17/75 (22.7)
PSA progression	16/75 (21.3)
**PSA RR at 90 days-**	
No of patient evaluable	29/42
≥50%	13/29 (44.8)
<50%	16/29 (55.2)


## Statistical analyses

**Figure 1:**
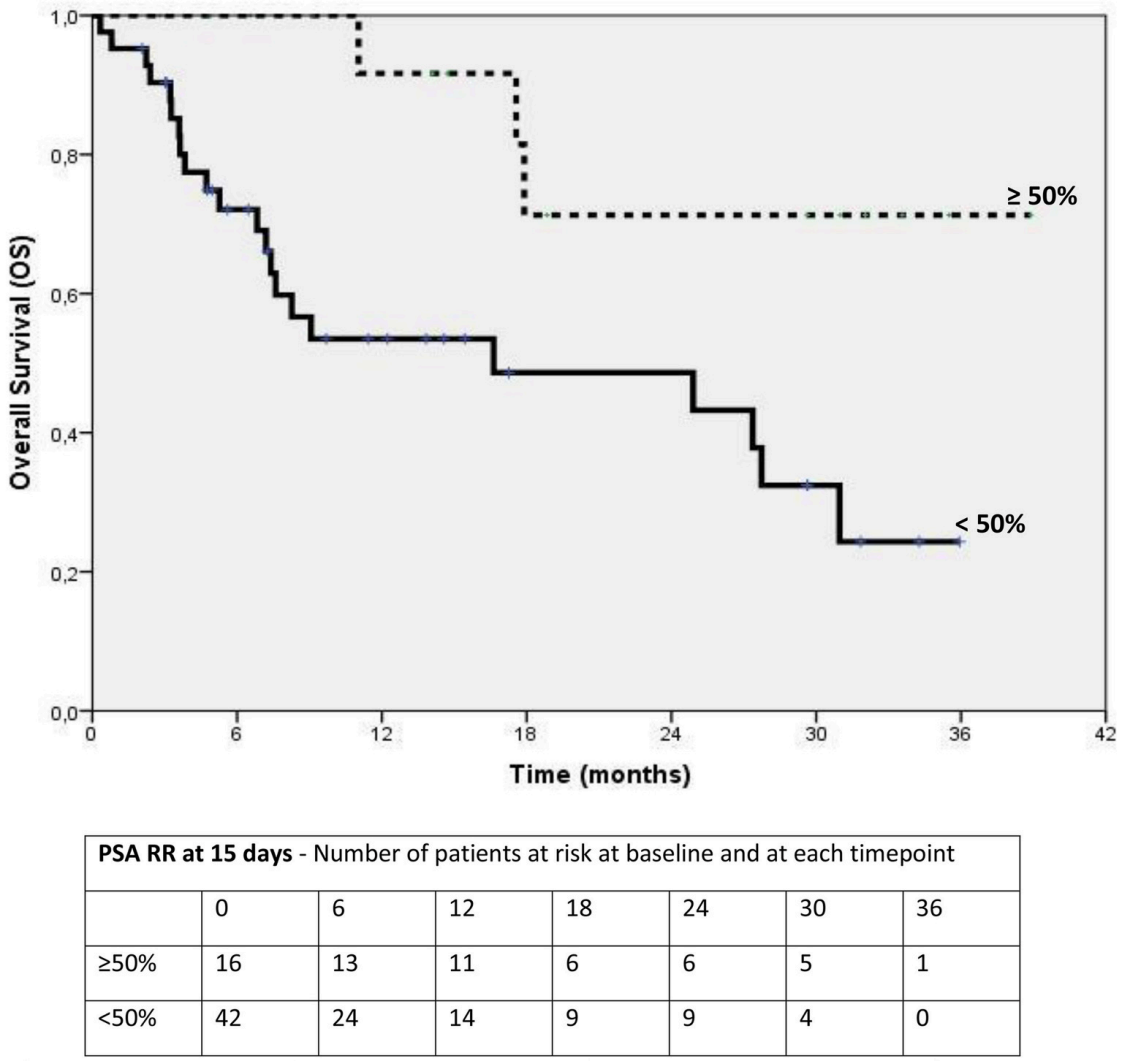
Kaplan-Meier estimates of Overall Survival according to PSA response rate* at 15 days. *Defined as the proportion of patients with a decrease of ≥50% in the PSA concentration from the basline PSA value to 15 days.

We record the time from the initial treatment with AA to progression as PFS; OS was defined as the time from the last follow-up to death. Univariate analysis for PFS and OS were calculated according to the Kaplan-Meier method. Statistical differences between curves were calculated using log-rank test. We analyzed PSA response rates computing a percentage-reduction at 15 days and 90 days through the following formula: [(a – b)/b^*^100] where a) baseline PSA; b) 15 days PSA or 90 days PSA; the proportion of patients with a decrease of ≥50% in the PSA concentration from the baseline PSA value were considered the best category. The Cox proportional hazards model was used to test the effect of PSA response rates on survival outcomes in multivariate analyses. Hazard ratios (HRs) and 95% CIs were estimated, adjusting for age and the PSA response rates were investigated as independent factors (Figure 1). Stratified analyses were carried out to assess whether PSA response rates were consistent across subgroups. A *p* < 0.05 was considered significant. Statistical analysis was performed using SPSS (version 21; SPSS, Inc., Chicago, IL).

## Results

**Figure 2:**
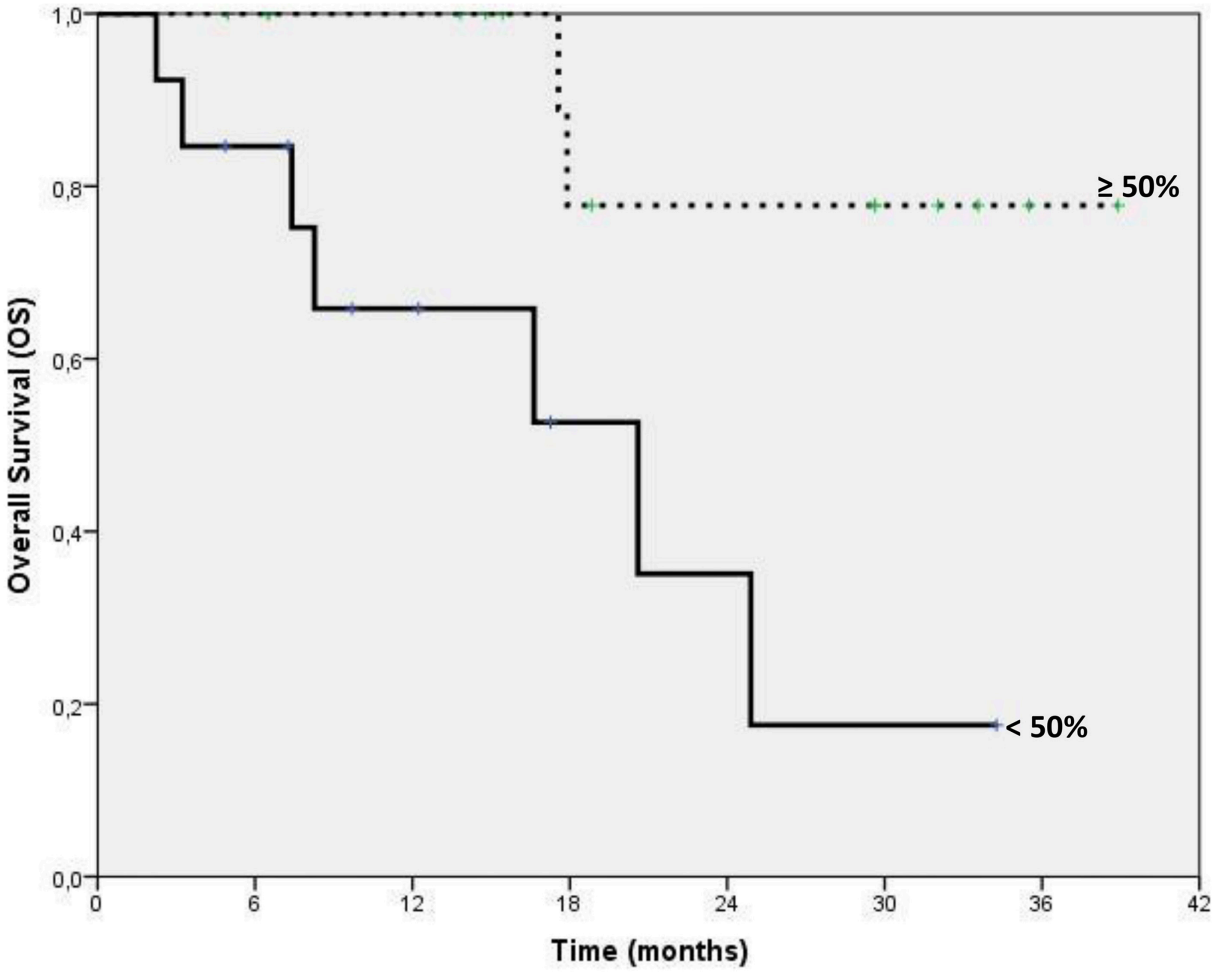
Kaplan-Meier estimates of Overall Survival according to PSA response rate at 90 days*. *Subgroup of 29 patients with confirmation of response obtained at 15 days included in the analysis of PSA response at 90 days.

Between Sep 2011 and Sep 2014, 87 patients' records were located, all had sufficient follow-up information and 75/87 were eligible for correlation analysis. The Table 1 summarize baseline patients' characteristics. Median serum PSA value at baseline was 332.2 ng/dl (range: 1–3922). PSA decline was observed in 78.6% (59/75) of patients. Early PSA response (≥50% from baseline at 15 days) was found in 56% (42/75) evaluated patients. At 90 days after starting AA, 29/42 patients confirm a PSA value of response. Confirmed PSA RR ≥ 50% was found in 31% (13/29). In early responders patients (PSA RR ≥ 50% at 15 days), we found a significant statistical advantage in terms of PFS at 1 year, HR 0.28, 95%CI 0.12–0.65, *p* = 0.003, and OS, HR 0.21 95% CI 0.06–0.72, *p* = 0.01 (Figure 1). The median OS was 17.1 months (8.8–25.2). In this group of early PSA responders, we evaluated the confirmed PSA RR at 90 days. The results in PFS at 1 years and OS (Figure 2) reached statistical significance, HR: 0.23 95% CI 0.07–0.77, *p* = 0.02 and HR: 0.14 95% CI 0.03–0.70, *p* = 0.02, respectively (Tables 2, 3). The proportion of patients alive at 1 year was 88% (*n* = 15) in PSA ≥ 50% and 12% (*n* = 5) in PSA < 50%; no patients were observed without progression at 1 year in both categories. Our analysis showed a positive correlation between OS and duration of AA treatment in early responders. Patients treated for more than 7 months with AA had a significant longer survival, HR 0.27 95% CI 0.13–0.57, *p* = 0.001.

**Table 3:** **Results of multivariate analysis in early PSA responders (≥50%)**.

**Variable**	**Median OS (months) and (95% CI)**	HR^*^	**(95% CI)**	***p*-value**
**ADVANTAGE IN OS**				
PSA RR at 15 days	16.6 (0.1–38.4)	0.21	0.06–0.07	0.01
PSA RR at 90 days	20.6 (6.4–34.7)	0.14	0.03–0.70	0.02
**Advantage in PFS at 1 year**	**Median PFS (months) and (95% CI)**			
PSA RR at 15 days	4.6 (2.3–6.9)	0.28	0.12–0.65	0.003
PSA RR at 90 days	5.6 (5.3–5.8)	0.23	0.07–0.77	0.02

* Multivariate Cox model adjusted for terms of age and center.

Patients with more than 70 years old had a longer OS, HR 0.03 95% CI 0.01–0.80, *p* = 0.032. The improvement in OS was achieved also in patients with one previous chemotherapy treatment, HR 0.11 95% CI 0.01–0.95, *p* = 0.045. Early responders who received a cumulative dose of docetaxel ≤675 mg/m^2^ confirmed a statistical advantage in OS, HR 0.07 95% CI 0.007–0.8, *p* = 0.032. Previous hormonal therapy duration (≥2.5 months) for metastatic setting correlated with longer OS, HR 0.06 95% CI 0.004–0.99, *p* = 0.05. These results are summarized in Tables 4, 5. Concerning the other stratification, we do not found a significant statistical correlation in terms of PFS and OS.

**Table 4:** **Univariate Analysis: median follow-up survival (Overall)**.

**Variables**	**Median (months)**	*p*-value^*^
**AA treatment duration**		<0.0001
<7 months	7.2	
≥7 months	27.7	
**Age**		0.5
<70 years	17.9	
≥70 years	12.4	
**Previous chemotherapy treatment**		0.4
≤1 line	3.5	
>2	7.2	
**Cumulative dose of Docetaxel**		0.8
≤675 mg/m^2^	17.06	
>675 mg/m^2^	16.6	
**Previous hormonal duration therapy**		0.5
<2.5 months	12.4	
≥2.5 months	17.5	

* Log-rank test.

**Table 5:** **Advantage in OS for early PSA RESPONDERS (≥50%)**.

**Variables**	HR^*^	**95% CI**.	***p*-value**
**AA TREATMENT DURATION**			
<7 months	r.c.		
≥7 months	0.27	0.13–0.57	0.001
**AGE**			
<70 years	r.c.		
≥70 years	0.03	0.01–0.80	0.032
**PREVIOUS CHEMOTHERAPY TREATMENT**			
≤1 line	0.11	0.01–0.95	0.045
>2	r.c.		
**CUMULATIVE DOSE OF DOCETAXEL**			
≤675 mg/m^2^	0.07	0.007–0.8	0.032
>675 mg/m^2^	r.c.		
**PREVIOUS HORMONAL DURATION THERAPY**			
<2.5 months	0.06	0.004–0.99	0.05
≥2.5 months	r.c.		

* Multivariate Cox model adjusted for age; r.c., reference category.

## Discussion

mCRCP is a heterogeneous disease in which AR remains activated and plays an important role during the progression of mCRPC. Several mechanisms contribute to continue AR signaling in mCRPC (Rathkopf and Scher, 2013). A significant percentage of patients with mCRPC that progresses in the course or after docetaxel are potential candidates to additional treatments in order to be able to increase OS (de Bono et al., 2011; Fizazi et al., 2012). Until few years ago, there were few therapeutic opportunities, formally recognized, with no outcome in terms of survival. To date there are at least three approved drugs such as AA, CBZ and ENZ (Bubley et al., 1999; de Bono et al., 2010, 2011; Fizazi et al., 2012; Scher et al., 2012; Bahl et al., 2013; Di Lorenzo et al., 2013).The most important question mark remains how to choose the best treatment sequencing and if there are some early response marker to guide clinical choice. To make effective use of secondary therapies there is a definite need to predict clinical or biological markers for early identification of non-responders patients, who may benefit from alternative treatments thus allowing a reduction in costs. All recent phase III trials have used as criteria for assessing the efficacy of some drugs, those established by the Prostate Cancer Working Group 2 (PCWG 2) criteria (Scher et al., 2008). These criteria define the efficacy of drugs in phase II trials and demonstrate whether the therapeutic effects observed justified further evaluation in large-scale phase III trials. These criteria do not reflect the clinical reality and are often difficult to apply. Many prognostic factors predict OS after docetaxel therapy (including pre-treatment variables such as bone pain, visceral metastases, performance status, anemia, bone scan progression, and PSA) but none of these variables is predictive of responses to new hormonal treatments (Armstrong and Febbo, 2009). Although several prostate cancer–associated antigens have been identified, PSA is the most commonly used (Miles et al., 2008). Many studies found that a PSA decline of > 50%, following chemotherapy, was highly prognostic because of is an early marker of drug activity and a potential surrogate of OS (Bubley et al., 1999; Petrylak et al., 2006; Armstrong et al., 2007a; Hussain et al., 2009; Caffo et al., 2012; Kijima et al., 2012). It is known that PSA can reflect the burden of disease in men with mCRPC. Decline in PSA value can reflect a reduction in disease burden and a potential clinical benefit with cytotoxic chemotherapy or hormonal agents (Vollmer et al., 1998; Scher et al., 1999; Armstrong et al., 2007b, 2012). There is a direct relationship between AR activity and PSA production. Hence, PSA could be a valid clinical marker to minimize patient exposure to ineffective therapy. Other retrospective studies based on clinical experience such as the Caffo et al. one (Caffo et al., 2014), aimed at identifying factors predicting primary resistance to new-generation hormonal agents. In this study, the role of these parameters was clearly demonstrated only in the cumulative analysis of patient treated with ENZ while in the AA group was not confirmed. Another study, presented at ASCO GU 2015 (Rescigno et al., 2015) conducted on 124 patients who had received AA post-docetaxel demonstrated that the PSA response of ≥30% at 4 weeks from the baseline was significantly correlated with OS (*P* < 0.001) in multivariate analyses including other established prognostic factors in mCRPC (ECOG PS, albumin, PSABL, ALP, LDH, Hemoglobin). In our study, the aim was to evaluate an early PSA RR, defined as ≥50% reduction from baseline at 15 days after started AA, and confirmed at 90 days (according PWCG2) as surrogate of increased PFS at 1 year and prolonged OS. In early responders patients we found a significant statistical advantage in terms of PFS at 1 year, HR 0.28, 95% CI 0.12–0.65, *p* = 0.003, and OS, HR 0.21 95% CI 0.06–0.72, *p* = 0.01. AA treatment lasting more than 7 months, one previous chemotherapy treatment, a cumulative dose of docetaxel ≤675 mg/m^2^ and previous hormonal therapy duration ≥2.5 years, correlated with longer OS.

A PSA expression correlated with the activation of AR. PSA decline seemed to be an excellent surrogate for survival in AR-driven tumor but not in AR-independent one. Docetaxel caused cytoplasmic AR sequestration *ex vivo* (Jiang and Huang, 2010; Zhu et al., 2010; Buonerba et al., 2011; Mezynski et al., 2012) and in circulating tumor cells (Darshan et al., 2011) with significant down-regulation of AR and PSA expressions, so the PSA value seemed not to be useful in CRPC AR-independent (Caraglia et al., 2005; Kuroda et al., 2009; Loriot et al., 2013; Caffo et al., 2015). Conversely, recent evidences have demonstrated the molecular heterogeneity of CRPC in terms of re-expression of the AR for genomic amplification, over-expression and up regulation after docetaxel treatment. The persistent activation of AR signal in all stages of CRPC has led to consider PSA decline as a valid predictive marker of treatment response and a surrogate of longer OS in apparently AR-independent CRPC (Armstrong et al., 2012; Rathkopf and Scher, 2013).

In any case the treatment with AA has been successfully continued even in those who did not register a reduction in early PSA with a median follow-up survival of 27.7 months in patients with a duration treatment of more than 7 months Probably this will lead us to distinguish patients in two groups with different sensitivity to the drug.

## Conclusion

In conclusion, these results have suggested that early PSA RR can provide clinically meaningful information and can be considered a surrogate of longer PFS and OS. In this context, early PSA RR may represent an early and easily obtainable predictive marker of response to AA. This is a retrospective analysis with a limited number of patients so further investigation with a larger cohort and prospective setting should be performed in the future.

## Author contributions

GF and SP design the study and wrote the manuscript. CDP collected data. AC performed the statistical analysis. OC, CO, CDA, MD, SC, FM, FR, GI, SP, MM, MB, and CC contributed to clinical data collection. All authors approved the manuscript.

### Conflict of interest statement

The authors declare that the research was conducted in the absence of any commercial or financial relationships that could be construed as a potential conflict of interest.
